# A Novel I221L Substitution in Neuraminidase Confers High-Level Resistance to Oseltamivir in Influenza B Viruses

**DOI:** 10.1093/infdis/jiu244

**Published:** 2014-05-03

**Authors:** Vanessa Escuret, Patrick J. Collins, Jean-Sébastien Casalegno, Sebastien G. Vachieri, Nicholas Cattle, Olivier Ferraris, Murielle Sabatier, Emilie Frobert, Valérie Caro, John J. Skehel, Steve Gamblin, Frédéric Valla, Martine Valette, Michèle Ottmann, John W. McCauley, Rodney S. Daniels, Bruno Lina

**Affiliations:** 1 Laboratoire de Virologie et Centre National de Référence virus influenzae; 2 Service de Réanimation Pédiatrique, Hôpital Femme Mère Enfant, Groupement Hospitalier Est,Hospices Civils de Lyon, Bron; 3 Laboratoire Virpath EA4610, Faculté de Médecine Lyon Est, Université Claude Bernard Lyon 1, Université de Lyon,and; 4 Genotyping of Pathogens and Public Health Platform, Institut Pasteur,Paris, France; 5 Division of Virology; 6 Division of Molecular Structure; 7 WHO Collaborating Centre for Reference and Research on Influenza, Division of Virology, Medical Research Council National Institute for Medical Research, London, United Kingdom

**Keywords:** influenza B virus, oseltamivir resistance, neuraminidase substitution I221L

## Abstract

Influenza B viruses with a novel I221L substitution in neuraminidase (NA) conferring high-level resistance to oseltamivir were isolated from an immunocompromised patient after prolonged oseltamivir treatment.

***Methods.*** Enzymatic characterization of the NAs (Km, Ki) and the in vitro fitness of viruses carrying wild-type or mutated (I221L) NA genes were evaluated. Proportions of wild-type and mutated NA genes were directly quantified in the patient samples. Structural characterizations by X-ray crystallography of a wild-type and I221L variant NA were performed.

***Results.*** The Km and Ki revealed that the I221L variant NA had approximately 84 and 51 times lower affinity for oseltamivir carboxylate and zanamivir, respectively, compared with wild-type NA. Viruses with a wild-type or I221L variant NA had similar growth kinetics in Madin-Darby canine kidney (MDCK) cells, and 5 passages in MDCK cells revealed no reversion of the I221L substitution. The crystal structure of the I221L NA and oseltamivir complex showed that the leucine side chain protrudes into the hydrophobic pocket of the active site that accommodates the pentyloxy substituent of oseltamivir.

***Conclusions.*** Enzyme kinetic and NA structural analyses provide an explanation for the high level of resistance to oseltamivir while retaining good fitness of viruses carrying I221L variant NA.

Influenza A and B viruses are important human pathogens. The neuraminidase inhibitors (NAIs) oseltamivir and zanamivir are the antiviral agents available in France to treat influenza A or B virus infections. Amantadine is ineffective against influenza B viruses, and influenza A viruses circulating since 2009 in humans are nearly all resistant to amantadine [1]. In 2007–2008, seasonal influenza A viruses bearing an H275Y substitution in neuraminidase (NA) conferring resistance to oseltamivir emerged in patients who were not receiving oseltamivir treatment [2]. However, most cases of influenza A or B viruses resistant to NAIs emerge in patients undergoing treatment, notably in children or immunocompromised patients [3–5].

The NA active site includes catalytic residues (R118, D151, R152, R224, E276, R292, R371, and Y406; N2 numbering) that interact directly with the sialic acid substrate and framework residues (E119, R156, W178, S179, D/N198, I222, E227, H274, E277, N294, and E425; N2 numbering) that stabilize the active site [6, 7]. NAs are divided into 3 phylogenic groups: influenza B viruses, group 1 (N1, N4, N5, and N8), and group 2 (N2, N3, N6, N7, and N9) from influenza A viruses [8]. Clinically relevant NA substitutions responsible for resistance of influenza viruses to NAIs, selected in vivo, usually map to specific framework residues and vary according to the NA subtype. The most frequent substitutions responsible for oseltamivir resistance in vivo correspond to H275Y [9], E119V/I [10–12], and D197N/E/Y [13, 14] for N1, N2, and influenza B virus neuraminidases, respectively. Influenza B viruses carrying NA-I221T and, more recently, the I221V substitution were recovered from untreated patients [15–18].

We are the first to report influenza B viruses, isolated from an immunocompromised patient after prolonged oseltamivir treatment, with good fitness carrying a novel I221L substitution (B numbering) in NA that confers high-level resistance to oseltamivir.

## MATERIALS AND METHODS

### Virological Diagnosis of Influenza Virus Infection

Nasopharyngeal aspirates (NPAs), bronchoalveolar lavage (BAL) samples, and nasal swab specimens were collected from an immunocompromised patient who had received prolonged oseltamivir treatment. Subsequently, virus culture medium was added to obtain a final volume of ≥1.5 mL. NPAs, nasal swab specimens, and BAL samples were screened for the presence of influenza virus, using a real-time reverse-transcription quantitative polymerase chain reaction (RT-qPCR; Influenza A/B r-gene, Argène) that can detect influenza A and influenza B viruses. RNA was extracted from 200 µL of sample, using the NucliSens easyMAG system (Biomerieux). Elution of the extracted nucleic acids was performed in 70 µL of the provided eluent. Respiratory samples were also cultured on Madin-Darby canine kidney (MDCK) cells to isolate virus; 2 or 3 passages were performed prior to performance of NA inhibition assays and genotypic analyses.

### NA Activity and Inhibition Assays

The NAIs zanamivir and oseltamivir carboxylate (GS4071) were kindly provided by GlaxoSmithKline and Roche, respectively. For each isolate, a fluorometric inhibition assay was performed in duplicate as described previously [19], except MES buffer (pH 6.4) was used. Briefly, total NA activities were calculated as the quantity of 2′-(4-methylumbelliferyl)-α-D-*N*-acetylneuraminic acid (MUNANA) substrate (Sigma) degraded to 4-methylumbelliferone (4-Mu) in 1 hour per mL of virus suspensions. The NA inhibition assay was then performed using a standardized amount of NA activity (10 nmol 4 Mu/h/mL) after dilutions of virus suspensions. The inhibitory concentrations (IC_50_) of oseltamivir and zanamivir, defined as the drug concentrations able to inhibit 50% of the NA activity, were calculated using Sigma Plot software. Interpretations of influenza B virus inhibition by NAIs are based on fold increases in IC_50_ values as compared to values for susceptible virus: normal inhibition was defined as <5-fold inhibition; reduced inhibition, as 5–50-fold inhibition; and highly reduced inhibition, as >50-fold inhibition [20].

### NA and Hemagglutinin Sequencing

Open reading frames for NA and hemagglutinin (HA) or HA1 were sequenced at the World Health Organization (WHO) collaborating center (London, United Kingdom) and the Institut Pasteur (Paris, France), using primers designed by the WHO collaborating center (sequences available on request). Sanger sequencing was performed on ABI Prism 3730XL DNA Analysers at the Institut Pasteur and the Medical Research Council National Institute for Medical Research (London, United Kingdom), with percentages of wild-type and mutant sequence variants estimated on the basis of sequence traces. Phylogenetic analyses of both genes showed the B/Lyon/CHU/2011 viruses (CHU is defined as “Centre Hospitalo-Universitaire” and denotes virus isolated from an hospitalized patient) recovered from the patient to be of the B/Victoria lineage (data not shown). The NA and HA glycoproteins showed limited amino acid variation, compared with that for the B/Brisbane/60/2008 reference strain (Supplementary Table 1), a recently recommended component of influenza vaccine, the NA structure of which was determined during the time of this study.

### Kinetic Analysis of NA Activities

The Michaelis-Menten constant (K_m_), which reflects the affinity of NA for the substrate, was evaluated in virus suspensions, using the MUNANA substrate as previously described [21]. The substrate was used at concentrations of 10 µM, 20 µM, 40 µM, 60 µM, 80 µM, 100 µM, 140 µM, and 200 µM. The 4-Mu fluorescence was measured each minute over 1 hour with a FLUOstar Optima fluorometer (BMG Labtech) at 37°C (λ excitation, 330 nm; λ emission, 450 nm). The initial velocity (V_i_) was calculated for each substrate concentration and integrated into a nonlinear Michaelis-Menten equation by the MARS program (BMG) to calculate K_m_. Each isolate was tested at least twice, and the results are means of the values obtained for each experiment.

The affinity of NA for an inhibitor (K_i_) was measured at drug concentrations of 0.01–10 nM for susceptible viruses and 50–3200 nM for resistant viruses. Because K_m_ values were between 6 µM and 12 µM, the substrate was used at a final concentration of 20 µM to obtain competitive inhibition under our experimental conditions. V_i_ was calculated for each inhibitor concentration and integrated into a nonlinear competitive inhibition equation by GraphPad software (Prism) to calculate K_i_.

### Cells

MDCK cells (CCL34; American Type Culture Collection, Manassas, VA) were maintained in serum-free medium (UltraMDCK; Lonza) supplemented with 1% L-glutamine (200 nM; Lonza) and 2% penicillin-streptomycin (PS; 10 000 U penicillin/mL and 10 000 U streptomycin/mL; Lonza).

### Plaque Assays

The first virus isolate with a 100% wild-type NA (B/Lyon/CHU/12.88/2011 (P2 MDCK)) and virus isolates that presented the highest oseltamivir IC_50_ with 100% I221L substituted NA (B/Lyon/CHU/15.216/2011 (P3 MDCK) and B/Lyon/CHU/16.167/2011 (P2 MDCK)) were selected for plaque assays. Six-well plates of confluent MDCK cells were incubated at 34°C for 1 hour with 10-fold serial dilutions of viruses in Eagle's minimal essential medium (EMEM; Lonza), 1% L-glutamine, and 2% PS supplemented with 1 µg/mL of trypsin (infection medium). The supernatants were then withdrawn, and the cells were overlaid with freshly prepared EMEM (diluted from a 2× concentrate) containing 0.55% agar (Agar Noble; Difco) and 1 µg/mL trypsin. We picked 5–10 plaques for each virus that were passaged individually on confluent MDCK cells in wells of 24-well plates. NA inhibition assays were then performed on virus culture supernatants to monitor the isolation of oseltamivir-susceptible or -resistant viruses. We then chose 1 virus isolate from each plaque assay for subsequent experiments and passages in MDCK cells. These virus isolates were named virus 1, virus 2, and virus 3, corresponding to viruses recovered from B/Lyon/CHU/12.88/2011 (P2 MDCK), B/Lyon/CHU/15.216/2011 (P3 MDCK), and B/Lyon/CHU/16.167/2011 (P2 MDCK), respectively.

### Infectious Titers

Infectious titers (ie, doses infecting 50% of the cell culture [TCID_50_]/mL) were determined as end-point-titration assays on confluent MDCK cells in 96-well plates. Briefly, 50 µL of serial 10-fold dilutions of each virus were inoculated into 4 replicate wells. The 96-well plates were incubated at 34°C for 4 days, and the presence of virus in supernatants was then evaluated by a hemagglutination assay, using guinea pig erythrocytes (0.8%). Infectious titers were calculated using the Reed and Muench statistical method [22].

### Passage in MDCK Cells

Virus culture supernatants for viruses 1, 2, and 3, recovered after plaque assays, were used to infect confluent MDCK cells in 24-well plates at a temperature of 34°C and a multiplicity of infection (MOI) of 10^−4^ TCID_50_/cell. Supernatants were harvested 3 days after infection and frozen at a temperature of −80°C. Infectious titers were determined as described above. Viruses were then passaged successively in MDCK cells 4 more times at an MOI of 10^−4^ TCID_50_/cell.

### Kinetics in MDCK Cells

Confluent MDCK cells (flask volume, 25 cm^2^) were infected at a MOI of 10^−3^ TCID_50_/cell. At the indicated times, 200 µL of supernatant was harvested (and replaced with infection medium warmed to a temperature of 34°C immediately after removal of the sample) and frozen at a temperature of −80°C. Infectious titers of each sample collected were determined as described above.

### Quantification of Viruses With a Wild-Type or I221L-Substituted NA in Clinical Specimens

A 1-step allelic discrimination real-time RT-qPCR was adapted for the studied influenza B viruses: the real-time RT-qPCR conditions were identical to conditions previously described for detecting the H275Y-conferring mutation in N1 genes [23]. Two probes specifically detect the presence of an isoleucine (Ile) or leucine (L) at position 221 of the NA for the studied influenza B viruses. Sequences of primers and probes are available on request. The sensitivity of this RT-qPCR was 1–10 copies for the detection of viruses bearing Ile or Leu at position 221 in NA, and it could detect the presence of at least 10% mutant (I221L) virus in a mixed-virus population.

### Detection of Mutated (I221L) NA Virus Subpopulations by NA Inhibition Assay of Virus Isolates

Mixtures of viruses propagated from plaque-purified B/Lyon/CHU/12.88/2011 and B/Lyon/CHU/16.167/2011 P2 MDCK isolates were created by increasing the amount of mutant in 10% increments until the complete mixture was mutant virus, on the basis of their relative NA activities, to obtain totals of mixed NA activity corresponding to fluorescence produced by 10 nmol of 4-methylumbelliferone/h/mL. The virus mixtures were analyzed by an NA inhibition assay. The apparent IC_50_ values for oseltamivir in the sample mixtures were plotted (using Sigma Plot) as a function of the mutant (I221L) concentration to obtain a nonlinear regression equation.

### Structural Analysis

Influenza B viruses B/Lyon/CHU/15.216/2011 (M3) and B/Brisbane/60/2008 (E4) were propagated in hens' eggs. The NA was released from virus by trypsin digestion and further purified, as described previously [24]. Crystals were obtained by adding 25% PEG 1500 and 0.1 M succinic acid (pH 9.0). Crystals were cryoprotected by the addition of 25% ethylene glycol, and 1 mM inhibitor made up in cryoprotectant was absorbed by the crystals. All crystals were frozen by direct immersion in liquid nitrogen, and diffraction data sets were collected at 100 K on the IO2 and IO4 beamlines at the Diamond light source (Harwell, United Kingdom). Data sets were indexed and integrated with XDS [25], imported in the CCP4 suite of programs [26] by using Pointless [27], and scaled and merged with Scala [27] or Aimless [28]. Structure amplitudes were calculated with Ctruncate [26]. Starting sets of phases were obtained by molecular replacement performed in Phaser [29], using B/Beijing/1/87 NA as the initial search model. Structures were subsequently built manually in Coot [30] and refined in Refmac [31] and phenix.refine and AutoBuild [32, 33]. Model quality was validated using MolProbidty [34]. Crystallographic statistics are summarized in Supplementary Table 2.

## RESULTS

### Clinical History of the Patient

The patient was a 17-year-old male who underwent liver transplantation at the age of 4 years because of a genetic deficiency of bile salt export pump protein. Several iterative graft rejections were identified by liver biopsy. The patient underwent several plasmapheresis sessions and was strongly immunocompromised by tacrolimus, mycophenolate, and corticosteroids.

**Figure 1.**
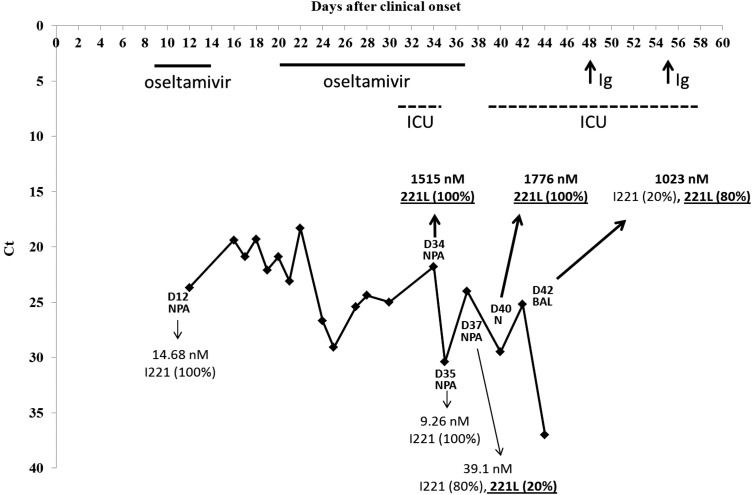
Virus load in clinical specimens during the course of influenza B virus infection. The real-time reverse-transcription quantitative polymerase chain reaction cycle threshold (Ct) is inversely proportional to the influenza virus load, and we present here an inverted *y*-axis to better represent viral load decrease during the course of influenza virus infection. The day (D) of sampling after clinical onset and the type of initial sample (nasopharyngeal aspirate [NPA], nasal swab specimen [N], and bronchoalveolar lavage [BAL] specimen) are indicated. Oseltamivir IC_50_ (nM) and the percentage of wild-type (I221) and mutated (221L) neuraminidase genes are indicated for each virus isolate. At the top of the graph, the duration of oseltamivir treatment, duration of intensive care unit (ICU) stay, and timing of immunoglobulins (Ig) administration are presented by bold lines, dotted lines, and arrows, respectively.

The patient presented with asthenia and cough on 9 March 2011 considered as day 0 (D0). Influenza B virus was first detected in a nasal swab specimen obtained on D8. Oseltamivir (75 mg orally, 2 times/day) was administered for 6 days (from D9 to D14), but NPAs were still positive for influenza B virus. Oseltamivir (75 mg orally, 2 times/day) was then administered again for 17 days (from D20 to D36). As NPAs were still positive for influenza B virus, oseltamivir treatment was stopped. The clinical history is summarized in Figure 1. The patient was admitted to an intensive care unit (where he stayed from D30 to D33) with liver, kidney, and brain failure and was readmitted to the ICU (where he stayed from D38 to D56) with liver failure. A new liver transplant was considered. To potentially enhance the patient's immune responses to influenza B virus, immunosuppression was gradually reduced from D41 by stopping plasmapheresis and lowering the doses of tacrolimus. Because the patient did not possess specific anti-influenza B virus immunoglobulins, treatment with intravenous immunoglobulins (which were positive for anti-influenza B virus immunoglobulins) was administered on D48 and D55. An NPA obtained on D50 tested negative for influenza B virus, as did those collected up to D72.

### Detection of Resistance to NAIs (IC_50_) and Enzymatic Characterization of NA (K_m_ and K_i_)

Viruses isolated in cell culture from D12 to D30 were susceptible to oseltamivir and zanamivir, with IC_50_ values of 14.7 nM and 2 nM, respectively, for the first isolate. Viruses isolated from an NPA collected on D34 and a nasal swab specimen collected on D40 were highly resistant to oseltamivir (IC_50_, 1515 and 1776 nM, respectively) and showed reduced inhibition by zanamivir (IC_50_, 24.3 and 27.3 nM, respectively), compared with the first wild-type isolate (Table 1). The K_m_ revealed that the I221L NA variant had a 1.9 times lower affinity for the MUNANA substrate, and the K_i_ values for oseltamivir carboxylate and zanamivir showed affinities that were around 84 and 51 times lower, respectively, than that for wild-type NA (Table 1).

**Table 1.** Neuraminidase (NA) Enzymatic Characterization of Influenza Viruses Isolated in Madin-Darby Canine Kidney Cells

Strain Designation^a^	Time After Clinical Onset, d	Sample Type	NA Activity^b^	IC_50_, nM^c^		K_m_, µM^c^	K_i_, nM^c^	
				Oseltamivir	Zanamivir		Oseltamivir	Zanamivir
B/Lyon/CHU/12.88/2011	12	NPA	263 ± 105	14.68 ± 10.40	2.14 ± 0.74	6.72 ± 0.28	5.85 ± 4.35	0.41 ± 0.06
B/Lyon/CHU/12.1127/2011	18	NPA	254 ± 1	12.05 ± 3.62	1.47 ± 0.49	8.07 ± 0.36	4.75 ± 3.23	0.45 ± 0.04
B/Lyon/CHU/13.584/2011	22	NPA	241 ± 59	11.65 ± 1.11	1.69 ± 0.01	8.51 ± 0.35	4.04 ± 1.91	0.41 ± 0.13
B/Lyon/CHU/14.411/2011	28	NPA	249 ± 35	12.53 ± 1.73	1.20 ± 0.18	9.18 ± 2.00	4.30 ± 1.98	0.50 ± 0.01
B/Lyon/CHU/14.767/2011	30	NPA	270 ± 19	9.85 ± 1.68	1.02 ± 0.18			
B/Lyon/CHU/15.216/2011	34	NPA	395 ± 87	**1515 ± 270 (103)**	**24.31 ± 1.84 (11)**	**12.78 ± 0.01 (1.9)**	**491.35 ± 64.70 (84)**	**20.85 ± 0.05 (51)**
B/Lyon/CHU/15.489/2011	35	NPA	282 ± 25	9.26 ± 5.74	1.33 ± 0.32	5.95 ± 0.40	4.01 ± 0.34	0.59 ± 0.06
B/Lyon/CHU/15.731/2011	37	NPA	289 ± 47	39.10 ± 8.16 (2.7)	1.93 ± 0.94	6.99 ± 1.15	7.60 ± 1.77	1.22 ± 0.03
B/Lyon/CHU/16.167/2011	40	N	365 ± 225	**1776 ± 10 (121)**	**27.25 ± 2.03 (13)**	**11.36 ± 1.41 (1.7)**	**472.85 ± 71.06 (81)**	**21.69 ± 0.98 (53)**
B/Lyon/CHU/16.432/2011	42	BAL	410 ± 81	**1023 ± 98 (70)**	**14.83 ± 2.13 (7)**	**11.85 ± 1.65 (1.7)**	**283.50 ± 22.20 (48)**	**8.81 ± 1.63 (21)**

Data presented are mean values (±SD) of 2 experiments.

Abbreviations: BAL, bronchoalveolar lavage; IC_50_, inhibitory concentration; K_i_, NA affinity for an inhibitor; K_m_, Michaelis-Menten constant; N, nasal swab; NPA, nasopharyngeal aspirate.

^a^ Names of influenza A viruses isolated from clinical specimens at different days after onset of symptoms

^b^ Data are nmol of 4-methylumbelliferone released per h and per mL of sample.

^c^ Determined using fluorometric assays, as described in Materials and Methods. Fold increases in IC_50_, Km, and Ki values compared with that of the first (B/Lyon/CHU/12.88/2011) wild-type virus are indicated in parentheses.

### Sequencing of NA and HA Genes

Sequencing of the NA gene in isolates revealed a unique mutation encoding an I221L substitution in NA at a level of 100% for isolates from a NPA obtained on D34 and a nasal swab specimen obtained on D40 and at a level of 80% for the isolate from a BAL obtained on D42. However, isolates from NPAs obtained on D35 and D37 bore wild-type NA at levels of 100% and 80%, respectively (Figure 1 and Supplementary Table 1). Analysis of HA and NA genes revealed that the only difference between oseltamivir-resistant and oseltamivir-susceptible isolates was the mutation conferring the I221L substitution in the NA gene (Supplementary Table 1).

### Detection of Mutated NA (I221L) in Clinical Specimens From the Patient

Some NA mutations responsible for reduced inhibition by NAI have been shown to arise during virus propagation in cell culture. One-step allelic discrimination real-time RT-qPCR was performed to assess the presence of the mutation conferring the I221L substitution in clinical specimens and determine whether the mutation was selected for and amplified during cell culture (Table 2).

**Table 2.** Estimated Proportion of Wild-Type (wt; I221) Neuraminidase (NA) in Clinical Specimens, Compared With the Oseltamivir Phenotype for the Corresponding Virus Isolate

Days After Clinical Onset	Type of Specimen	NA Gene Level, Copies/mL Specimen^a^	wt (I221) NA Proportion, %	Virus Recovered	Oseltamivir Phenotype
12	NPA	280 000	100	B/Lyon/CHU/12.88/2011	NI (wt)
18	NPA	4 760 000	100	B/Lyon/CHU/12.1127/2011	NI (wt)
22	NPA	6 090 000	100	B/Lyon/CHU/13.584/2011	NI (wt)
28	NPA	217 000	100	B/Lyon/CHU/14.411/2011	NI (wt)
30	NPA	770 000	100	B/Lyon/CHU/14.767/2011	NI (wt)
34	NPA	4 172 000	1	B/Lyon/CHU/15.216/2011	HRI (221L)
35	NPA	6300	100	B/Lyon/CHU/15.489/2011	NI (wt)
37	NPA	3 920 000	11	B/Lyon/CHU/15.731/2011	NI (wt)
40	N	259 000	5	B/Lyon/CHU/16.167/2011	HRI (221L)
42	BAL	4 018 000	0.70	B/Lyon/CHU/16.432/2011	HRI (221L)

Quantification of the wt or mutant NA was performed using real-time reverse-transcription quantitative polymerase chain reaction, using specific probes for I221 (wt) or 221L (mutant) NA, as described in Materials and Methods.

Abbreviations: BAL, bronchoalveolar lavage; N, nasal swab; NPA, nasopharyngeal aspirate.

^a^ Data total no. of I221 (wt) and 221L (mutant) NA gene copies.

The quantification of wild-type or mutated NA genes in clinical specimens from the patient revealed good correlations with viruses recovered in cell culture: 99% and 95% of mutated NA gene being detected in clinical specimens obtained on D34 and D40, respectively, and 100% of wild-type NA gene being detected in clinical specimens obtained on D35. However, the proportions of the mutated NA gene were 89% and 99% in clinical specimens obtained on D37 and D42, but only 20% and 80%, respectively, in the corresponding isolates (Table 2 and Figure 1), suggesting a possible partial selection for wild-type virus by MDCK cells for these samples.

### Impact on Fitness

**Figure 2.**
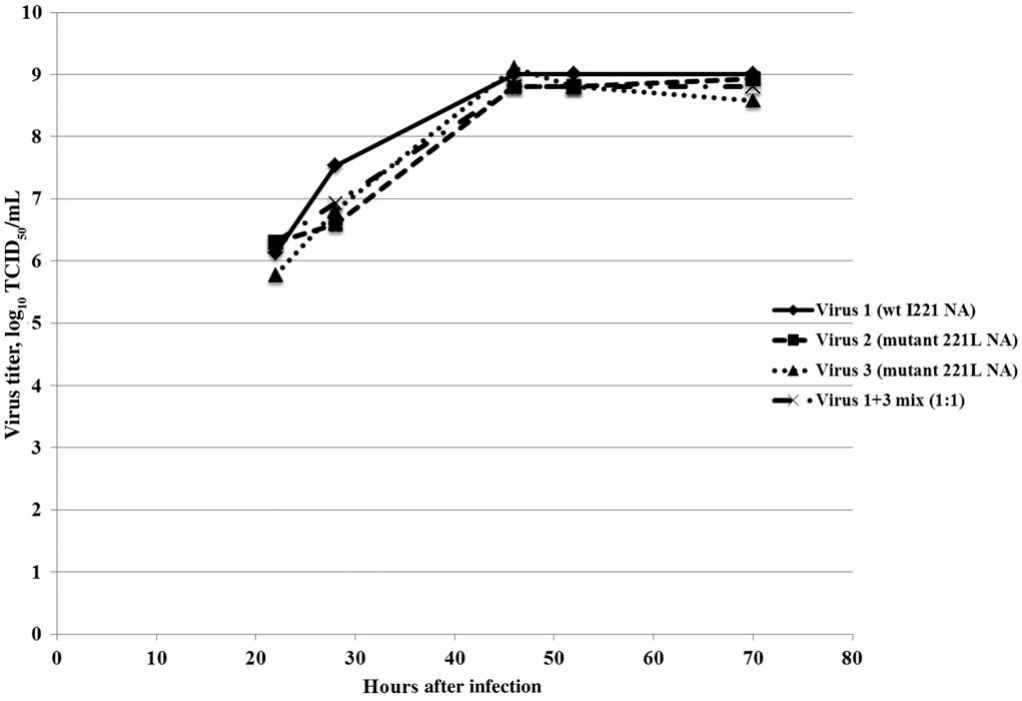
Growth kinetics in Madin-Darby canine kidney (MDCK) cells of influenza B viruses bearing a wild-type (wt; I221) or mutant (221L) neuraminidase (NA). Virus growth kinetics were assessed under 1-step conditions, using MDCK cells with the same passage history. Confluent MDCK cells were infected at a multiplicity of infection of 10^−3^ by virus 1, virus 2, or virus 3 or a 1 + 3 mix. At 0, 4, 22, 28, 46, 52, and 70 hours after infection, 200 µL of supernatants were harvested and titrated as described in Materials and Methods. Abbreviation: TCID_50_, tissue culture infective dose.

Virus kinetics experiments performed under 1-step conditions showed wild-type viruses and mutated NA (I221L) viruses to have similar growth kinetics in MDCK cells (Figure 2). Five passages of wild-type and mutated NA (I221L) viruses in MDCK cells (MOI, 10^−4^) with no selective pressure revealed the stability of viruses bearing wild-type or mutated NA (data not shown) and neither gain nor reversion of the I221L substitution (Table 3).

**Table 3.** Persistence of Mutant (221L) Neuraminidase (NA) After 5 Passages of Viruses in Madin-Darby Canine Kidney (MDCK) Cells

Initial Virus Isolate (MDCK Cell Passage)	Virus Name After Plaque Assay^a^	MDCK Passage^b^	NA Activity^c^	IC_50_, nM^d^	
				Oseltamivir	Zanamivir
B/Lyon/CHU/12.88/2011 (P2)	Virus 1 (wt [I221] NA)	P4	90	5.82	1.91
		P4 + P1	54	10.16 ± 0.83	ND
		P4 + P5	55	8.97 ± 0.29	ND
B/Lyon/CHU/15.216/2011 (P3)	Virus 2 (mutant [221L] NA)	P5	68	1245	29.06
		P5 + P1	53	1257 ± 88	ND
		P5 + P5	43	1251 ± 93	ND
B/Lyon/CHU/16.167/2011 (P2)	Virus 3 (mutant [221L] NA)	P4	58	1699	49.04
		P4 + P1	63	1190 ± 86	ND
		P4 + P5	53	1419 ± 121	ND

Abbreviations: IC_50_, inhibitory concentration; ND, no data; wt, wild-type.

^a^ Viruses 1, 2, and 3 were each recovered after 1 passage in MDCK cells of their respective parental virus, B/Lyon/CHU/12.88/2011 (P2 MDCK), B/Lyon/CHU/15.216/2011 (P3 MDCK), and B/Lyon/CHU/16.167/2011 (P2 MDCK), as described in Materials and Methods.

^b^ Viruses 1, 2, and 3 were cultured for 1 (+P1) and 5 (+P5) more passages, from the initial recovered isolate, in triplicate in MDCK cells.

^c^ Data are nmol of 4-methylumbelliferone released per h and per mL of sample.

^d^ Determined for virus culture supernatants.

To better understand the viral fitness of viruses bearing a wild-type or mutated NA (I221L), we performed competition experiments by mixing (1:1) viruses propagated from the plaque assays of the first wild-type isolate and the isolate showing the highest oseltamivir-resistance. We then performed passages in MDCK cells in triplicate and determined IC_50_ values on culture supernatants to estimate the proportion of wild-type or mutated NA (Table 4). This experiment revealed that in 2 of 3 cases, the resistant isolate rapidly became dominant in the virus population, demonstrating that the resistant virus was not significantly less fit than the wild-type virus in culture.

**Table 4.** Proportion of Wild-Type (wt; I221) or Mutant (221L) Neuraminidase (NA) in Virus Populations After 5 Passages of a 1:1 Virus Mixture

Initial Virus Isolate (MDCK Cell Passage)	Virus Name After Plaque Assay^a^	MDCK Passage^b^	NA Activity^c^	IC_50_, nM^d^		Estimation of Mutant (221L) NA, %^e^
				Oseltamivir	Zanamivir	
B/Lyon/CHU/12.88/2011 (P2)	Virus 1 (wt [I221] NA)	P4	90	5.82	1.91	<0
B/Lyon/CHU/16.167/2011 (P2)	Virus 3 (mutant [221L] NA)	P4	58	1699.00	49.04	>100
	Virus 1 + 3 mix (1:1) (a)	P4 + P1	68	19.22	ND	19.51
		P4 + P2	54	8.79	ND	<0
		P4 + P3	56	8.77	ND	<0
		P4 + P4	67	9.46	ND	<0
		P4 + P5	65	10.66	ND	0.91
	Virus 1 + 3 mix (1:1) (b)	P4 + P1	68	682.30	ND	82.1
		P4 + P2	43	1078.00	ND	>100
		P4 + P3	72	1309.00	ND	>100
		P4 + P4	70	1247.00	ND	>100
		P4 + P5	70	1238.00	ND	>100
	Virus 1 + 3 mix (1:1) (c)	P4 + P1	80	68.22	ND	44.12
		P4 + P2	66	115.40	ND	52.83
		P4 + P3	59	940.10	ND	92.26
		P4 + P4	100	1101.00	ND	>100
		P4 + P5	79	852.80	ND	87.83

Abbreviations: IC_50_, inhibitory concentration; MDCK, Madin-Darby canine kidney; ND, no data.

^a^ Viruses 1 and 3 recovered from plaque assays of B/Lyon/CHU/12.88/2011 and B/Lyon/CHU/16.167/2011 P2 MDCK isolates, respectively, were mixed at an equal tissue culture infective dose TCID_50_/mL (mix 1:1).

^b^ Up to 5 passages (+P1 to +P5) of the virus 1 + 3 mix (1:1) were performed in triplicate in MDCK cells.

^c^ Data are nmol of 4-methylumbelliferone released per h and per mL of sample.

^d^ Determined for virus culture supernatants.

^e^ Oseltamivir IC_50_ values were plotted according to an established nonlinear regression equation, as explained in Materials and Methods, to estimate the proportion of wt (I221) or mutant (221L) NA.

### Structural Analysis

**Figure 3.**
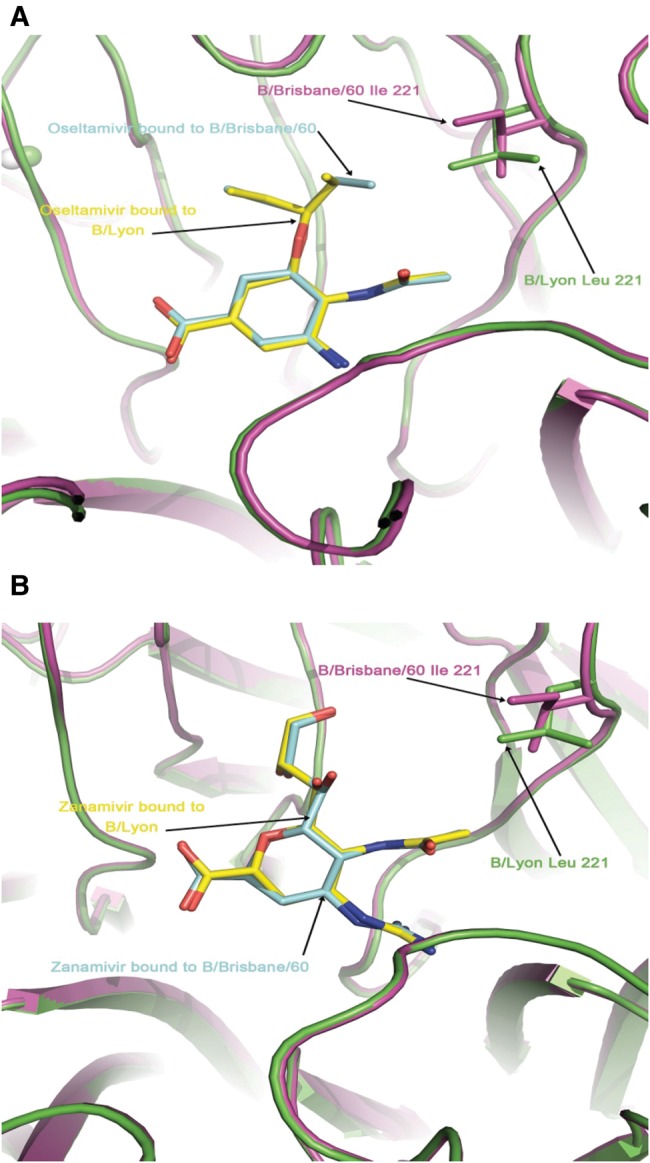
Overlaid structures of the active sites of wild-type (purple; B/Brisbane/60/2008) and mutant (I221L; green; B/Lyon/CHU/15.216/2011) neuraminidase with bound inhibitors oseltamivir (*A*) and zanamivir (*B*).

We used X-ray crystallography to understand the structural basis of resistance to oseltamivir conferred by NA carrying the I221L substitution. Crystals of the NA-I221L protein in complex with both inhibitors diffracted to high resolution, showing clearly defined density for bound oseltamivir and zanamivir (Supplementary Table 2). The structure of the NA-I221L-oseltamivir complex showed that the L side chain protrudes into the hydrophobic pocket of the active site that accommodates the pentyloxy substituent of oseltamivir, thus causing a change in the conformation of the inhibitor such that its C81 carbon moves about 2.2 Å from the wild-type NA bound position (Figure 3*A*). Consistent with lower levels of inhibition of the I221L mutant virus by zanamivir, no marked changes in the conformation of zanamivir were seen (Figure 3*B*).

## DISCUSSION

I221 (B NA numbering), I222 (N2 numbering), and I223 (N1 numbering) amino acids are highly conserved framework residues in NAs of influenza viruses [7]. Different substitutions at these positions have been described in vivo for influenza A or B viruses, with different impacts in terms of levels of resistance to NAI. This article contains the first description of an I221L substitution in the NA gene of influenza B viruses. Mutation causing this substitution emerged in an immunocompromised patient treated with oseltamivir; it confers highly reduced inhibition by oseltamivir, defined according to recent recommendations on interpretation of influenza virus susceptibility [20, 35], without any marked loss of virus fitness (ie, the capacity of a virus to produce infectious progeny in a given environment [36]). Influenza B viruses carrying the NA I221T substitution have been previously isolated in the course of global surveillance studies [16, 17] and in patients before treatment [15]. This substitution confers 3-, 7-, or 13-fold increases in the oseltamivir IC_50_, with values of 10.48 nM, 513.8 nM, and 100 nM according to the respective studies. More recently, a cluster of 14 influenza B viruses carrying the NA I221V substitution, present as a mixed population, were isolated in North Carolina (from November 2010 through February 2011) in untreated patients [18]. The mean oseltamivir IC_50_ of the influenza B viruses bearing an NA I221V/I polymorphism was 19.86 nM (a 2-fold increase, compared with corresponding wild-type influenza B viruses) [37]. The level of resistance of the I221V substitution might be higher because the wild-type virus propagated in tissue culture more readily than the I221V mutant, as assessed by pyrosequencing of clinical specimens and virus isolates [18]. In comparison to the I221T or I221V substitutions, the I221L substitution emerged in an immunocompromised patient who underwent prolonged oseltamivir treatment and conferred an increase of >100-fold in the oseltamivir IC_50_, a much higher fold increase than those seen for the other substitutions.

Substitutions at position NA I223 (I223/T/V/L/K/F/M; N1 numbering) have been described in vivo in avian influenza A(H5N1) viruses, conferring variable increases in IC_50_ [38–40]. Of 782 NA sequences from influenza A(H5N1) viruses, 24 had substitutions at NA 223: I223T (15 strains), I223V (4 strains), I223L (2 strains), and I223K/F/M (1 each) [40]. The I223L substitution identified in A/chicken/Laos/P0020/2007 was associated with K150N and S246N substitutions and contributed to a 77-fold increase in the mean oseltamivir IC_50_ (84.3 nM) [41]. For clade 2.1 A(H5N1) viruses, the oseltamivir IC_50_ values ranged from 43 nM to 75 nM for the I223T/V substitutions and from 268 nM to 349 nM for the I223M substitution [39]. In experimental infections of ferrets with influenza A(H5N1) viruses, the I223L substitution emerged spontaneously without any drug pressure [42].

Influenza A(H1N1) viruses with an I223V substitution alone have been detected during global influenza surveillance (eg, A/Hamamatsu/92/2002) [16] and associated with the H275Y substitution during the 2009 influenza A(H1N1) pandemic in immunocompetent patients under oseltamivir prophylaxis [9]. Influenza A(H1N1)pdm09 viruses carrying a NA I223R substitution conferring a 46-fold increase in oseltamivir IC_50_ (9.1 nM) were isolated from an immunocompromised patient treated with intravenous zanamivir [43]. Influenza A(H1N1)pdm09 isolates carrying both I223R and H275Y substitutions were isolated from immunocompromised patients after prolonged oseltamivir and/or zanamivir treatment and were highly resistant to oseltamivir [44, 45]. Characterization of reassortant A(H1N1)pdm09 viruses showed that the NA I223V substitution alone conferred only a slight increase in oseltamivir IC_50_ but induced a synergistic increase in oseltamivir IC_50_ when associated with the H275Y mutation (1733 nM vs 982 nM for H275Y alone). Interestingly, viruses carrying the NA I223V substitution propagated to significantly higher titers than wild-type virus and could restore virus fitness and partially compensate for the loss of NA activity due to the H275Y substitution [46]. Conversely, for reassortant viruses generated by reverse genetics, the NA I223R substitution appears to reduce virus titers [45]. In the present study, influenza B viruses bearing a wild-type or I221L-substituted NA showed similar in vitro replicative fitness.

Substitutions at position I222V (N2 numbering) have also been described in patients undergoing treatment for influenza A(H3N2) virus infections [10]. As for the I223V/R and H275Y substitutions in N1, the N2-I222V substitution alone confers a slight increase in oseltamivir IC_50_ but acts in synergy with E119V (in N2) to yield highly reduced inhibition by oseltamivir [10, 47].

In summary, in all published cases where NA-I222 substitution (N2 numbering) occurs alone, virus fitness is not impaired, and only mild increases in oseltamivir IC_50_ are observed. In our case, I221L substitution in NA of influenza B virus isolates induced highly reduced inhibition by oseltamivir, with IC_50_ values greater than concentrations of oseltamivir achievable in body fluids (ie, sinus fluid, middle ear fluid, and plasma) under single and multiple dosing regimens [48]. Moreover, the NA I221L substitution does not impair virus replicative fitness. Taken together, influenza B viruses carrying the I221L substitution in NA can be considered clinically resistant, thereby presenting significant difficulties in the management of infection.

In conclusion, this study shows that the management of influenza virus infections can be difficult in immunocompromised patients. Efforts to better understand the immune response against influenza virus infections and to improve vaccines and therapeutic options are needed.

## Supplementary Data

Supplementary materials are available at The Journal of Infectious Diseases online (http://jid.oxfordjournals.org). Supplementary materials consist of data provided by the author that are published to benefit the reader. The posted materials are not copyedited. The contents of all supplementary data are the sole responsibility of the authors. Questions or messages regarding errors should be addressed to the author.

Supplementary Data
