# Supplementary material for: A Novel I221L Substitution in Neuraminidase Confers High-Level Resistance to Oseltamivir in Influenza B Viruses
Source: J Infect Dis. 2014 May 3;210(8):1260–9. doi: 10.1093/infdis/jiu244 (PMC4176448; doi:10.1093/infdis/jiu244)
Supplement: Supplementary Data [file supp_210_8_1260__index.html]

A Novel I221L Substitution in Neuraminidase Confers High-Level Resistance to Oseltamivir in Influenza B Viruses — A Novel I221L Substitution in Neuraminidase Confers High-Level Resistance to Oseltamivir in Influenza B Viruses — Supplementary Data 

# A Novel I221L Substitution in Neuraminidase Confers High-Level Resistance to Oseltamivir in Influenza B Viruses

## Supplementary Data

Supplementary Data

**Files in this Data Supplement:**

- Supplementary Data - Doc file
