# Supplementary material for: A Novel I221L Substitution in Neuraminidase Confers High-Level Resistance to Oseltamivir in Influenza B Viruses
Source: J Infect Dis. 2014 May 3;210(8):1260–9. doi: 10.1093/infdis/jiu244 (PMC4176448; doi:10.1093/infdis/jiu244)
Supplement: Supplementary Data [file supp_jiu244_jiu244supp.doc]

**Table S1. HA and NA sequencing data for virus isolates**

Amino acid substitutions in NA and HA compared to B/Brisbane/60/2008 sequences are indicated: amino acids in equivalent proportions (=), one amino acid in greater proportion than the other (at least 60 : 40% >), one amino acid in significantly greater proportion than the other (at least 80 :20% >>) based on outputs from Sanger sequencing.

^a^ E represents passage in Eggs, M represents passage in MDCK cells

^b^ Sequences were uploaded to the GISAID (Global Initiative on Sharing All Influenza Data) database.

^c^ Amino acid abbreviations: A (Alanine), D (Aspartic Acid), E (Glutamic Acid), F (Phenylalanine), I (Isoleucine), K (Lysine), L (Leucine), N (Asparagine), P (Proline), S (Serine), T (Threonine), V (Valine)

^d^ Amino acid substitutions at these positions are associated with loss of glycosylation at position 197 on adaptation to growth in hens’ eggs.

^e^ Viruses that provided NA for structural studies.

**Table S2. Crystallographic data collection and refinement statistics**

Brisb/08 = NA isolated from B/Brisbane/60/2008 (E4/E1); Lyon/11 = NA isolated from B/Lyon/CHU/15.216/2011 (M3/E1). Structures were determined for the NAs alone (Apo) or with oseltamivir (Osel) or zanamivir (Zana) in complex. The six structures have been deposited in the Protein Database (4CPL, 4CPM, 4CPN, 4CPO, 4CPY, 4CPZ).

^a^ Statistics for the highest-resolution shell are shown in parentheses.

^b^ Twin fraction as refined using Refmac 5 Twin refinement option, based on intensities; na: not applicable.
